# Evaluation of a Novel Detection Method for Allergen-Specific IgE Antibodies with IgE Receptor Crosslinking Using Rat Food Allergy Model

**DOI:** 10.3390/foods13172713

**Published:** 2024-08-27

**Authors:** Soichiro Ishii, Yuki Koga, Tomoharu Yokooji, Misaki Kakino, Ryohei Ogino, Takanori Taogoshi, Hiroaki Matsuo

**Affiliations:** 1 Department of Pharmaceutical Services, Graduate School of Biomedical and Health Sciences, Hiroshima University, Hiroshima 734-8551, Japan; sishii@hiroshima-u.ac.jp (S.I.); ykoga@hiroshima-u.ac.jp (Y.K.); yokooji@hiroshima-u.ac.jp (T.Y.); taogo@hiroshima-u.ac.jp (T.T.); 2 Department of Frontier Science for Pharmacotherapy, Graduate School of Biomedical and Health Sciences, Hiroshima University, Hiroshima 734-8553, Japan; b155364@hiroshima-u.ac.jp (M.K.); ryogino@hiroshima-u.ac.jp (R.O.)

**Keywords:** allergen-specific IgE, amplified luminescence proximity homogeneous assay, crosslinking, food allergy, gluten, high-affinity IgE receptors, ovalbumin

## Abstract

The specific detection of serum IgE antibodies specific to allergens (sIgE Abs) that can crosslink the plural high-affinity IgE receptor (FcεRIα) molecules on the surface of mast cells or basophils with a multivalent allergen can reduce the false-positive diagnoses observed in chemiluminescent and fluorescence enzyme immunoassays for type-I allergic patients. In this study, we detected sIgE Abs to the egg-allergen ovalbumin (OVA) and the wheat-allergen gluten in the sera of rats sensitized with each allergen using an amplified luminescence proximity homogeneous assay by crosslinking (AlphaCL). OVA and gluten were reacted with each sIgE Ab in the sera. Then, acceptor and donor beads labeled with the human FcεRIα were added to the reacted solution. The luminescence intensity for anti-OVA IgE Abs in the sera with the removal of IgG Abs was observed in five of seven (71.4%) of the sensitized rats, whereas no signals were observed in any of the unsensitized rats. The AlphaCL could also detect anti-gluten sIgE Abs in the sera of sensitized rats, but not of unsensitized rats. In conclusion, we successfully detected sIgE Abs in the sera of rats sensitized to two allergens using the AlphaCL. This detection method has the potential to be used as a new diagnostic tool for type-I allergic patients.

## 1. Introduction

Food allergy is an adverse reaction triggered by allergen-specific immunological mechanisms following exposure to the causative food. The prevalence of food allergy has been increasing, mainly in countries where Western diets are more prevalent [1]. The prevalence of food allergy among preschool children in developed countries has reached 8% [2]. Patients with food allergy suffer from a variety of allergic symptoms in the skin, mucous membranes, digestive tract, and respiratory tract. In addition, patients with food allergy may experience life-threatening anaphylaxis [3]. The mortality rate was estimated at 0.03–0.32 for food-induced anaphylaxis [4].

The symptoms of food allergy are typically elicited by type-I allergic reactions. These are triggered by chemical mediators such as histamine released from mast cells and basophils when plural allergen-specific IgE (sIgE) antibodies (Abs) bound to high-affinity IgE receptors (FcεRI) on the cell surfaces are crosslinked by a multivalent allergen [5,6]. The detection of serum sIgE Abs can be used for the screening of causative allergens in type-I allergic patients [7,8,9]. Fluorescence enzyme immunoassays (FEIAs) such as ImmunoCAP™ (Thermo Fisher Scientific, Waltham, MA, USA) are clinically used to measure serum sIgE levels in type-I allergic patients. However, FEIAs may give false-positive diagnoses because they detect total serum sIgE Abs regardless of the crosslinking ability [10,11,12]. Unlike the FEIAs, the basophil activation test (BAT) and histamine release test (HRT) can only detect sIgE Abs with crosslinking ability. However, their results are strongly affected by the activity of the patient’s basophils after collection [13,14]. Patients with food allergies should avoid the ingestion of causative foods to prevent the development of allergic symptoms. Therefore, false-positive diagnoses can reduce the quality of life for patients and their families through the unnecessary elimination of foods. Furthermore, false-negative diagnoses can lead to a risk of anaphylaxis by the accidental ingestion of causative foods for patients because patients believe that they are not allergic to suspected foods [15,16,17]. Therefore, there is a need for a simple diagnostic tool for food allergy with fewer false-positive and false-negative results in clinical settings.

**Figure 1:**
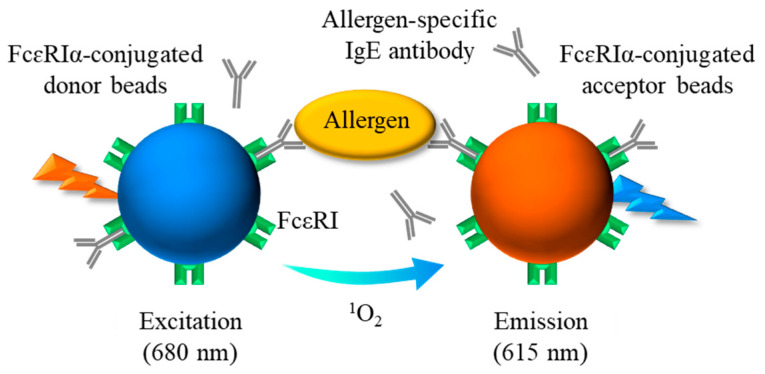
Schematic diagram showing the principle of AlphaCL for detection of sIgE Abs. When the sIgE Abs bound to FcεRIα on each bead are brought into proximity by crosslinking with the allergen, the acceptor beads can receive singlet oxygen emitted from the donor beads by excitation at 680 nm. A sharp emission peak at 615 nm is due to the energy generated by the singlet oxygen within the acceptor beads. sIgE, allergen-specific IgE; FcεRI, high-affinity IgE receptors.

We previously established an assay to detect functional IgG autoantibodies with a crosslinking ability against FcεRIα and IgE Abs in the sera of patients with autoimmune chronic spontaneous urticaria using an amplified luminescence proximity homogeneous assay by crosslinking (AlphaCL) [18]. Using the AlphaCL, we also established an assay model to detect serum sIgE Abs (Figure 1) [19]. The AlphaCL can reduce the false-positive diagnoses observed in FEIAs because this assay can specifically detect sIgE Abs that can crosslink the plural FcεRI with a multivalent allergen. However, in our previous report, the signal counts for sIgE detection in simulated human serum were decreased to <1% of those in the buffer [18]. We speculate that this is presumably due to the disruption of sIgE binding to FcεRI proteins on the bead surfaces by free non-specific IgE (nsIgE) and IgG Abs, which are highly abundant in the serum. Furthermore, we have not confirmed that the AlphaCL detects sIgE Abs in the sera of type-I allergic patients, because this assay model was established using 2,4-dinitrophenyl (DNP)-conjugated bovine serum albumin (BSA) as a simulated allergen and anti-DNP sIgE Abs as sIgE Abs. In this study, we attempted to examine the effect of IgG removal on the sensitivity for the detection of sIgE detection in the serum using the AlphaCL. We also aimed to detect sIgE Abs to the egg-allergen ovalbumin (OVA) and the wheat-allergen gluten in the sera of rats sensitized with each allergen.

## 2. Materials and Methods

### 2.1. Animals

Male 3-week-old Brown–Norway rats were obtained from Japan SLC (Shizuoka, Japan). All experiments involving animals were conducted following the guidelines for animal experiments set by the Hiroshima University Animal Experiment Facility Committee (Hiroshima, Japan). This study was approved by the Hiroshima University Animal Ethics Committee (Approval number: A-21-148-2).

### 2.2. Sensitization Protocol

The sensitization to OVA or gluten was performed according to our previous methods with a slight modification [20]. Briefly, rats were immunized by intraperitoneal injection with 0.5 mL of physiological saline (0.9% NaCl) containing 4 mg/mL of OVA (Sigma-Aldrich, St. Louis, MO, USA) and Imject^®^ Alum [5 mg/mL of Al(OH)_3_ and 5 mg/mL of Mg(OH)_2_] (Thermo Fisher Scientific) at weekly intervals for 2 weeks. Unimmunized rats received physiologic saline containing the adjuvant alone at the same schedule. For gluten, rats were immunized by subcutaneous injection with 0.5 mL of physiological saline containing 5 mM acetic acid, 2 mg/mL of commercial gluten (Tokyo Chemical Industry, Tokyo, Japan) and Imject^®^ Alum [10 mg/mL of Al(OH)_3_, and 10 mg/mL of Mg(OH)_2_] once. Unimmunized rats received physiologic saline containing acetic acid and adjuvant alone at the same schedule. After immunization with gluten or vehicle, inactivated Bordetella pertussis cells (6 × 10^8^ cells) were administered intraperitoneally. Four weeks after the initial immunization with OVA or gluten, blood was collected from the abdominal aorta and centrifuged to collect the serum.

### 2.3. Detection of Serum Anti-OVA and Anti-Gluten sIgE Abs by ELISA

To confirm the sensitization to OVA and gluten, the serum levels of anti-OVA or anti-gluten sIgE Abs were measured by ELISA according to our previous method, with slight modifications [20]. Each well of the plate (F8 MaxSorp loose Nunc-Immuno™ Modules, Thermo Fisher Scientific) was coated with 1 µg/mL of anti-rat IgE (MARE-1, GeneTex, Irvine, CA, USA), dissolved in phosphate-buffered saline (PBS, 100 µL, pH 7.4), and incubated overnight at 4 °C for solid-phase immobilization. Each well was washed with PBS containing 0.1% Tween 20 (PBS-T). Then, the plate was incubated with 1% Block Ace^®^ (100 µL, DS Pharma Biomedical, Osaka, Japan) for 1 h at 37 °C. Each well was washed with PBS-T, and then each serum sample (100 μL, diluted 1:10 with 10% Block Ace^®^) was added to the wells. After 1 h of incubation at room temperature (for anti-OVA sIgE Abs) or 2 h of incubation at 37 °C (for anti-gluten sIgE Abs), each well was washed with PBS-T. Then, the wells were incubated with 100 μL of 1% Block Ace^®^ containing OVA (diluted 1:500) or gluten (diluted 1:1000) labeled with biotin using a commercial kit (Biotin Labeling Kit-NH_2_, Dojindo, Kumamoto, Japan) for 1 h at room temperature (for anti-OVA sIgE Abs) or 2 h at 37 °C (for anti-gluten sIgE Abs). Each well was washed with PBS-T and incubated with horseradish peroxidase-conjugated streptavidin (100 µL, Proteintech Japan, Tokyo, Japan) for 1 h at room temperature (for anti-OVA sIgE Abs) or 37 °C (for anti-gluten sIgE Abs). Each well was washed with PBS-T and incubated with 3,3’,5,5’-tetramethylbenzidine (100 µL, SeraCare Life Sciences, Gaithersburg, MD, USA) for 15 min at room temperature (for anti-OVA sIgE Abs) or at 37 °C (for anti-gluten sIgE Abs). To terminate the reaction, 100 µL of 1 M phosphoric acid was added. Absorbance was measured at 450 nm against a reference of 620 nm using a Multiskan GO spectrophotometer (Thermo Fisher Scientific). These experiments were conducted twice.

### 2.4. Effect of IgG Removal on the Detection of sIgE in Serum by AlphaCL

The AlphaCL was performed according to our previous report, with slight modifications [19]. Donor and acceptor beads were conjugated with recombinant human FcεRIα proteins (R&D SYSTEMS, Minneapolis, MN, USA) by covalent bonding between the carboxyl group on the beads and the amino group of FcεRIα proteins, according to the manufacturer’s protocol (each 5 mg/mL), (Perkin Elmer, Waltham, MA, USA). Each bead was diluted with Alpha Immunoassay buffer (Perkin Elmer) and mixed at the appropriate concentration.

To evaluate the effect of serum IgG Abs on the detection of sIgE Abs, anti-DNP IgE Abs in human serum or anti-OVA sIgE Abs in rat serum were detected following the removal of IgG Abs using a protein G HP SpinTrap™ column (Cytiva, Tokyo, Japan). Briefly, anti-DNP sIgE Abs (YAMASA, Chiba, Japan) were added to a commercial human-pooled serum at a concentration of 0.1 μg/mL (Sigma-Aldrich, St. Louis, MO, USA). This sample and the sera of rats unsensitized or sensitized to OVA were diluted 1:1 with Alpha Immunoassay buffer (Perkin Elmer), respectively. Then, each sample was divided into “untreated” and “column-treated” groups. The samples in the column-treated group were processed on the column according to the manufacturer’s protocol. Each sample (20 μL) was mixed with DNP-BSA (0.05 μg/mL, 20 μL) for the detection of anti-DNP sIgE Abs or OVA (1 μg/mL, 20 μL) for the detection of anti-OVA sIgE Abs in a plate (1/2 Area Plate-96 plate, Perkin Elmer). After 1 h of incubation at room temperature, a bead mixture solution (10 μL; acceptor beads, 100 μg/mL; donor beads, 25 μg/mL) was added. After 24 h of incubation at room temperature in the dark, Alpha signals were counted by a multi-mode plate reader (NIVO™, Perkin Elmer). Net counts were calculated from the total counts in each sample by subtracting the counts in the buffer without sIgE Abs. These experiments were conducted in duplicate. The cut-off value was set as the mean plus two-fold standard deviations (SDs) of buffer alone.

### 2.5. Optimization of Detection for Anti-OVA sIgE Abs in Rat Serum by AlphaCL

The most appropriate serum dilution, OVA concentration, and the incubation time were determined to optimize the detection of anti-OVA sIgE Abs. OVA (0.004–2 μg/mL, 20 μL) and OVA-sensitized rat serum (#2), treated through a column (25–100%, 20 μL), were incubated for 1 h at room temperature in a 1/2 Area Plate-96 plate. Then, 10 μL of the bead mixture solution was added and incubated for 1–48 h at room temperature in the dark. To determine the detection limit of serum anti-OVA sIgE levels in AlphaCL, OVA-sensitized rat serum (#2) was diluted to various concentrations with commercial normal rat serum (Fujifilm Wako Pure Chemical Corporation, Osaka, Japan). These samples were further diluted at a 1:1 ratio with the buffer and processed on the column. The samples (1.56–50%, 20 μL) and OVA (1 μg/mL, 20 μL) were added to the plate. After 1 h of incubation at room temperature, 10 μL of the bead mixture solution was added and incubated for 24 h at room temperature in the dark. The Alpha signals were counted and calculated using the same method described above. These experiments were conducted in duplicate.

### 2.6. Measurement of the Serum Anti-OVA sIgE Abs Titer

The anti-OVA sIgE titer in the OVA-sensitized rat serum (#2) was determined using the LBIS^®^ mouse anti-OVA IgE assay kit according to the manufacturer’s protocol (Fujifilm Wako Pure Chemical Corporation, Guangzhou, China).

### 2.7. Detection of Anti-OVA and Anti-Gluten sIgE Abs in Sera of Rats Sensitized with Each Allergen by AlphaCL

For the detection of anti-OVA sIgE Abs, each serum sample from rats unsensitized (*n* = 3) or sensitized to OVA (*n* = 7) was processed on the column. Each sample (20 μL) was incubated with OVA (1 μg/mL, 20 μL) in the plate for 1 h at room temperature. For the detection of anti-gluten sIgE Abs, 20 μL of each column-treated serum sample from rats unsensitized (*n* = 3) or sensitized to gluten (*n* = 3) was incubated with gluten (0.1 μg/mL, 20 μL) for 1 h at room temperature. After 24 h of incubation with 10 μL of the bead mixture solution, the signals were counted by a multi-mode plate reader. These experiments were conducted twice.

### 2.8. Statistical Analysis

The data are presented as the mean ± SD. Differences in mean counts between groups were evaluated using the Student’s *t*-test and the Tukey–Kramer test. The strength of correlation between the results of ELISA and AlphaCL was analyzed using Spearman’s rank correlation coefficient. A *p*-value < 0.05 was considered statistically significant.

## 3. Results

### 3.1. Detection of Anti-OVA and Anti-Gluten sIgE Abs Levels in Rat Serum by ELISA

To confirm the sensitization to OVA or gluten, anti-OVA or anti-gluten sIgE Abs levels in the serum were determined using ELISA (Table 1). The serum levels (optical density 450 nm) of anti-OVA sIgE Abs in rats immunized with OVA (0.43 ± 0.16) were significantly higher than those in unimmunized rats (0.0005 ± 0.0002, *p* < 0.01). The higher serum levels of anti-gluten sIgE Abs were also observed in rats immunized with gluten (0.25 ± 0.06) compared to those in unimmunized rats (0.0006 ± 0.0002, *p* < 0.01). These results suggested that rats immunized with OVA or gluten were sensitized to each allergen.

### 3.2. Effect of IgG Abs Removal on the Detection of sIgE Abs in Human and Rat Serum by AlphaCL

**Figure 2:**
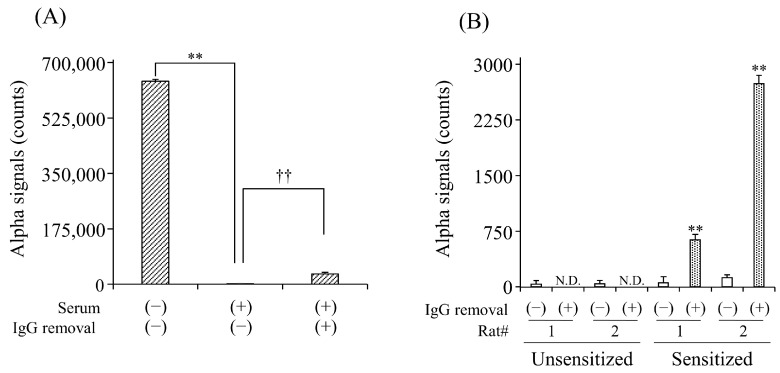
Effect of IgG removal from serum on the detection for serum anti-DNP (**A**) and anti-OVA (**B**) sIgE Abs by AlphaCL. Diluted human serum containing anti-DNP sIgE Abs and sera of rats unsensitized and sensitized to OVA were processed on the protein G HP SpinTrap™ column. Net counts were calculated from the total counts in each sample by subtracting the counts in buffer without sIgE Abs. ** *p* < 0.01 was considered significantly different from buffer solution. ^††^ *p* < 0.01 was considered significantly different from serum without IgG removal. All experiments were conducted in duplicate. N.D., not detected.

To evaluate the effect of serum IgG Abs on the detection of sIgE Abs, we first compared the detection of anti-DNP IgE Abs in the simulated serum with and without the removal of IgG Abs. In the untreated serum, signal counts were significantly lower than those in the buffer solution (Figure 2A). The column treatment significantly increased the counts by 18.3-fold compared to the non-treatment. We further examined the effect of IgG removal on the detection of anti-OVA IgE Abs in the sera of OVA-sensitized rats. In unsensitized rats, no signal counts were observed in the serum with or without column treatment. In contrast, the column treatment significantly increased the counts in the serum of rats sensitized to OVA compared to the non-treatment (Figure 2B).

### 3.3. Determination of the Optimal Serum Dilution, OVA Concentration, and Incubation Time for the Detection of Anti-OVA sIgE Abs in Rat Serum by AlphaCL

**Figure 3:**
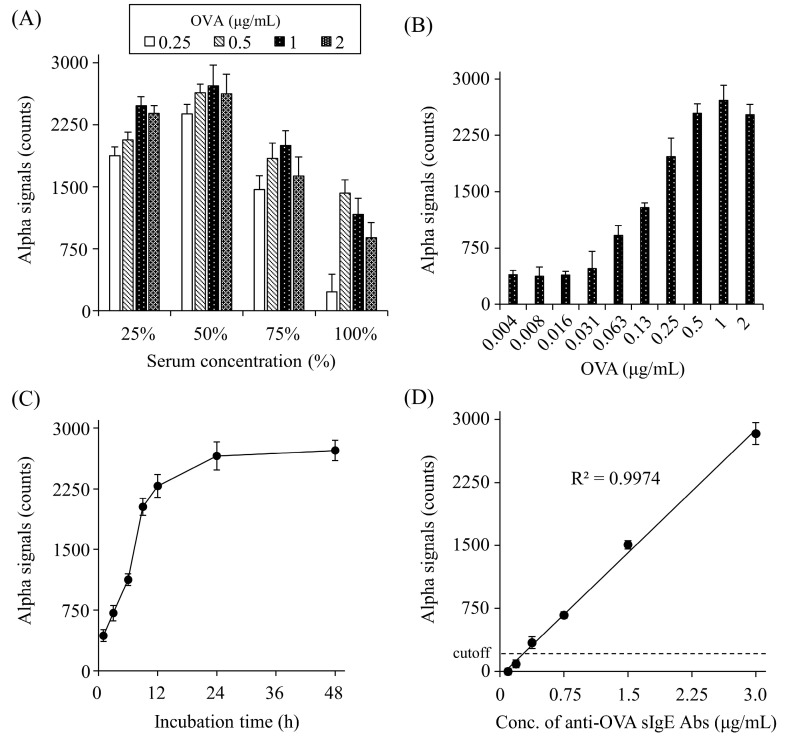
Optimization of serum dilutions (**A**), OVA concentrations (**B**), and the incubation time (**C**), and concentration-dependent crosslinking of each bead by serum anti-OVA sIgE Abs (**D**). Net counts were calculated from the total counts in each sample by subtracting the counts in buffer without sIgE Abs. The cut-off value was set as the mean plus two standard deviations of buffer alone. All experiments were conducted in duplicate.

To optimize the detection of anti-OVA sIgE Abs, the appropriate serum dilution, O VA concentration, and incubation time were determined. The signal counts increased gradually up to 50% serum and decreased at higher concentrations (Figure 3A). OVA caused a gradual increase in the signal counts up to 1 μg/mL and plateaued at higher concentrations (Figure 3B). As for the incubation time of OVA and serum, signal counts gradually increased until 24 h and then plateaued (Figure 3C).

### 3.4. Determination of the Detection Limit for Anti-OVA sIgE Abs in Rat Serum by AlphaCL

We determined the detection limit for anti-OVA sIgE Abs in rat serum using the AlphaCL. In this analysis, we used the serum of a rat sensitized with OVA (#2), which was diluted to various concentrations with normal rat serum. The ELISA results showed that the non-diluted serum (100%) from this rat contained 5.9 μg/mL of anti-OVA sIgE Abs. In the AlphaCL, the signal counts decreased linearly with the serum dilution, and the signal counts were below the cut-off value at concentrations <6.25%. Therefore, 0.37 μg/mL of anti-OVA sIgE Abs in 6.25% serum was set as the detection limit (Figure 3D).

### 3.5. Detection of Anti-OVA and Anti-Gluten sIgE Abs in Sera of Rats Sensitized to Each Allergen

**Figure 4:**
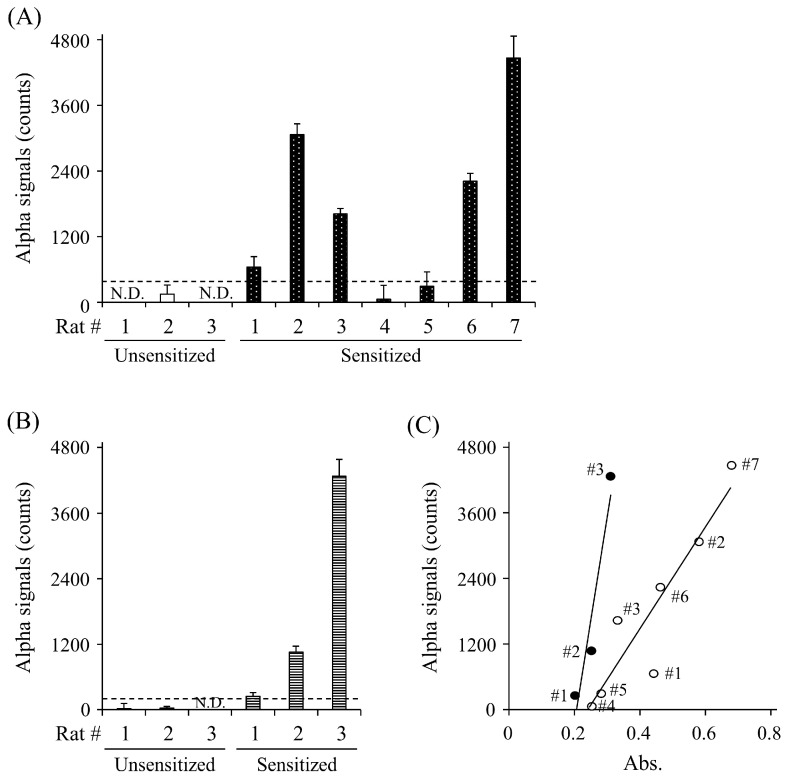
Detection of anti-OVA sIgE Abs (**A**) and anti-gluten IgE Abs (**B**) in sera of rats unsensitized (open column) and sensitized (closed column) to OVA (*n* = 7) and gluten (*n* = 3) by ELISA and AlphaCL. The scatter plots of serum sIgE levels of sensitized rat to OVA (open circle) and gluten (closed circle) detected between ELISA and AlphaCL (**C**). Statistical correlations were obtained by Spearman’s correlation coefficient and rank test. Net counts were calculated from the total counts in each sample by subtracting the counts in buffer without sIgE Abs. All experiments were conducted in duplicate. Dashed lines represent cut-off values. N.D., not detected.

To evaluate whether the AlphaCL is useful for the specific detection of sIgE Abs in rats sensitized to food allergens, we attempted to detect anti-OVA and anti-gluten sIgE Abs in the sera of rats sensitized to each allergen. The signal counts for anti-OVA IgE Abs were observed in five of the seven (71.4%) sensitized rats, whereas no signals were observed in any of the unsensitized rats (Figure 4A). The AlphaCL could also detect anti-gluten sIgE Abs in the sensitized rats, but not the unsensitized rats (Figure 4B). A strong correlation (r = 0.964) between the concentrations of anti-OVA sIgE Abs measured by ELISA (absorbance) and the AlphaCL signal counts in the OVA-sensitized rats was observed (Figure 4C).

## 4. Discussion

The AlphaCL can reduce the false-positive diagnoses observed in the enzyme immunoassay because this assay can specifically detect sIgE Abs that can crosslink the plural FcεRI with a multivalent allergen, though conventional FEIAs detect total serum sIgE Abs regardless of crosslinking ability. However, the sensitivity for sIgE detection in serum was remarkably lower than that in the buffer solution [19]. In this study, we succeeded in improving the sensitivity for the detection of serum sIgE Abs in the AlphaCL by removing serum IgG Abs using the protein G column. We also demonstrated that the AlphaCL could specifically detect sIgE Abs in the sera of rats sensitized to OVA or gluten. Therefore, AlphaCL may be useful to detect functional sIgE Abs that trigger symptoms in type-I allergic patients. In addition, the AlphaCL is superior to conventional cellular assays in the following ways: (1) it can be easily performed in facilities with fluorescent plate readers, (2) it does not require the use of basophils or cultured mast cells, (3) it is minimally invasive to the patient, and (4) it is interchangeable for a variety of allergens, as discussed previously [18].

The sensitivity for the detection of anti-DNP sIgE Abs in the serum by the AlphaCL was significantly lower than that in the buffer solution (Figure 2A). It has been reported that anti-OVA IgG Abs as well as sIgE Abs were increased in OVA-sensitized rats [21]. In general, IgG Abs in human [22] and rat [23] serum is considered to be more than 10,000-fold higher than IgE Abs, including sIgE Abs. Therefore, we speculated that the bindings of sIgE Abs with an allergen might be disturbed by the high concentration of serum IgG Abs. In support of this speculation, the signal counts for the detection of serum sIgE Abs were significantly increased by removing IgG using the protein G column compared to the non-treatment. (Figure 2B). However, the elevated signal counts for the detection of anti-DNP sIgE Abs in the serum due to IgG removal were only 18.3-fold greater than that in the untreated serum, whereas their counts in the untreated serum was less than 0.3% of that in the buffer solution with the same anti-DNP sIgE Ab concentration (Figure 2A). This result indicates that there may be other serum components as well as IgG Abs that interfere with the detection of sIgE Abs. The reason for the significant reduction in the signal count in the serum is unclear. However, we speculate that the binding of sIgE Abs to FcεRIα proteins on the beads might be interfered with by free nsIgE Abs in the serum. Furthermore, our preliminary study showed that signal counts for the detection of serum anti-DNP sIgE Abs were increased by a treatment with an ultrafiltration column with a molecular cut-off weight of 100 kDa. We also speculate that serum components with less than 100 kDa may also interfere with the detection, because this column cannot remove the IgE and IgG Abs. For example, the free-radical scavenging component in the serum can capture the singlet oxygen that was transferred from the donor beads to the acceptor beads [24].

We determined a 50% serum concentration, 1 μg/mL of OVA, and 24 h incubation time as the optimal conditions for the detection of serum anti-OVA sIgE Abs using the AlphaCL. In addition, our preliminary study showed that 0.1 μg/mL of gluten was appropriate for the detection of anti-gluten sIgE Abs. For the detection of anti-OVA sIgE Abs, the signal counts were decreased when the serum concentration exceeded 75% (Figure 3A). This result may be due to interference with detection by serum components, including IgG Abs unremoved by the column treatment. OVA caused a gradual increase in the signal counts up to 1 μg/mL and plateaued at higher concentrations (Figure 3B). This result may be explained by the fact that the formation of the OVA and anti-OVA sIgE Abs complex was saturated because the small amounts of anti-OVA sIgE Abs are insufficient for those of OVA. The signal counts showed a peak at 24 h of incubation. This result agrees with our previous report that signal counts were exhibited to be the highest at 24 h of incubation for the detection of anti-DNP IgE Abs in human serum [19]. Cellular assays such as BAT and HRT take only 4–6 h to produce results. Therefore, diagnostic tools with long incubation times might be disadvantageous when an early diagnosis is needed. In this study, we did not compare serum sIgE levels between healthy subjects and type-I allergic patients. If signal counts are significantly greater in patients than in healthy subjects in a short period of time, it can be conducted in less than 24 h. Under this optimal condition, the detection limit of serum anti-OVA sIgE Abs in AlphaCL was calculated as 0.37 μg/mL (Figure 3D). For type-I allergic patients, a serum level of sIgE Abs ≥ 0.0084 is considered positive using ImmunoCAP™ [22]. Although sIgE titers cannot be directly compared between rats and humans, the sensitivity should be further increased for the clinical use of AlphaCL as a diagnostic tool.

Although anti-OVA sIgE Abs could be detected in OVA-sensitized rats #4 and #5 by ELISA, the Alpha signal could not be obtained in them (Table 1 and Figure 4A). In addition to ImmunoCAP™, ELISA detects total anti-OVA sIgE Abs, including the sIgE Abs unable to crosslink IgE receptors. Therefore, we speculate that rats #4 and #5, which were sensitized to OVA, may have no functional anti-OVA sIgE Abs with a crosslinking ability. In this study, we did not evaluate the functional anti-OVA sIgE Abs by HRT, BAT, or allergic symptoms in the provocation test. Further studies are necessary to confirm the correlation between functional serum sIgE levels detected using the AlphaCL and these tests. In addition to purified anti-OVA sIgE Abs, the AlphaCL could detect anti-gluten sIgE Abs in the sera of rats sensitized, but not in the sera of unsensitized rats. This result suggests that the AlphaCL could be available to screen for various allergens in type-I allergic patients.

This study has several limitations. First, we could not remove serum components, including nsIgE and other Abs, such as IgA Abs and IgM Abs in the serum. High levels of free nsIgE Abs in the serum may inhibit the binding of sIgE to the FcεRIα protein on the bead surfaces. Furthermore, IgA Abs, IgM Abs, and IgG Abs may competitively inhibit the binding of sIgE Abs to the allergen if the serum contains the allergen-specific Abs that recognize the same binding epitope as the sIgE Abs. Second, we did not evaluate the functional anti-OVA sIgE Abs by HRT, BAT, or allergic symptoms in the provocation test in the allergen-sensitized rats. In particular, the improvement of sensitivity for the detection of serum sIgE Ab detection is an urgent issue to be addressed.

## 5. Conclusions

In this paper, we improved the sensitivity for the detection of serum sIgE Abs using the AlphaCL by removing IgG Abs. We also successfully detected sIgE Abs in serum samples from rats sensitized to two food allergens using the AlphaCL. Because the AlphaCL method is convenient and practical, it has the potential to be used as a new diagnostic tool for type-I allergic patients.

## Figures and Tables

**Table 1:** Determination of serum levels of IgE Abs specific to OVA and gluten.

	No.	ELISA (A.U.)		*p*-Value
OVA		Unsensitized	Sensitized	
	1	0.0007	0.44	
	2	0.0004	0.58	
	3	0.0005	0.33	
	4	–	0.25	
	5	–	0.28	
	6	–	0.46	
	7	–	0.68	
	Mean ± SD	0.0005 ± 0.0002	0.43 ± 0.16	0.0019
Gluten		Unsensitized	Sensitized	
	1	0.0006	0.20	
	2	0.0005	0.25	
	3	0.0008	0.31	
	Mean ± SD	0.0006 ± 0.0002	0.25 ± 0.06	0.0014

Serum levels of IgE Abs specific to OVA and gluten in unsensitized and sensitized rats were determined by ELISA. *p* < 0.05 was considered statistically significant. OVA, ovalbumin.

## Data Availability

The original contributions presented in the study are included in the article, further inquiries can be directed to the corresponding author.

## Abbreviations

Ab, antibody; AlphaCL, amplified luminescence proximity homogeneous assay; BAT, basophil activation test; BSA, bovine serum albumin; DNP, 2,4-dinitrophenyl; FcεRI, high-affinity IgE receptor; FEIA, fluorescence enzyme immunoassay; HRT, histamine release test; nsIgE, non-specific IgE; OVA, ovalbumin; PBS, phosphate-buffered saline; PBS-T, PBS containing 0.1% Tween 20; sIgE, allergen-specific IgE.
